# Palladium/Zirconium Oxide Nanocomposite as a Highly Recyclable Catalyst for C-C Coupling Reactions in Water

**DOI:** 10.3390/molecules15074511

**Published:** 2010-06-24

**Authors:** Antonio Monopoli, Angelo Nacci, Vincenzo Calò, Francesco Ciminale, Pietro Cotugno, Annarosa Mangone, Lorena Carla Giannossa, Pietro Azzone, Nicola Cioffi

**Affiliations:** 1 Department of Chemistry, Università degli Studi Aldo Moro, Via Orabona 4, 70126-Bari, Italy; 2 CNR – ICCOM, Department of Chemistry, Università degli Studi Aldo Moro, Via Orabona 4, 70126-Bari, Italy

**Keywords:** C-C coupling, palladium, colloids, green chemistry, nanocomposite catalysts

## Abstract

Palladium nanoparticles have been electrochemically supported on zirconium oxide nanostructured powders and all the nanomaterials have been characterized by several analytical techniques. The Pd/ZrO_2_ nanocatalyst is demonstrated to be a very efficient catalyst in Heck, Ullmann, and Suzuki reactions of aryl halides in water. The catalyst efficiency is attributed to the stabilization of Pd nanophases provided by tetra(alkyl)-ammonium hydroxide, which behaves both as base and PTC (phase transfer catalyst) agent.

## 1. Introduction

In the last decade, metal nanoparticles have attracted considerable attention in catalysis: their unique properties, intermediate between those of the bulk and the single particles, combine the advantages of heterogeneous catalysis (recovery and recyclability) with those of homogeneous catalysis (low loadings and good selectivity)[1,2]. Among all transition metals palladium [3], is certainly the one capable of promoting the widest range of reactions. In this context, palladium nanoparticles have been reported to be very active and selective in a great number of processes, including hydrogenations, C–C couplings and oxidations [4,5].

These nanocatalysts are generally prepared from a metal salt and a reducing agent, in the presence of stabilizing agents such as ligands [6] and surfactants [7], to prevent their aggregation. This approach provides stability to the nanoparticles formed, but in many cases the strong absorption of stabilizers on the active sites may diminish the catalytic activity, and moreover this method doesn’t solve the problems related to recovery and recycling of the expensive catalyst, which is a task of great economic and environmental importance in the industry.

An alternative method for generating highly recyclable nanocatalysts is the use of supports capable of anchoring nanoparticles strongly, leaving the active sites well dispersed and easily accessible on their surface. In principle, a support has to be physically and chemically stable during the reaction process, it must assure stability to the catalyst, its easy removal from the reaction mixture and reusability with minimal loss of catalytic activity. Moreover, the nature of the support can strongly affect the catalyst properties and activities as well as the particle size, structure, and methods of preparation of the composite. To this end charcoal [8], dendrimers [9], organic polymers [10], metal oxides [11], clays [12] and silica [13] have been used over the last few years.

Based on this approach, we recently reported the preparation of a nanocomposite material formed by electrochemical impregnation of nanostructured tetragonal ZrO_2_ with palladium nanoparticles (Pd-NPs/ZrO_2_) [14]. Our method of anchoring the catalyst on the surfaces of a nano-powder left a good surface area of the catalyst particles exposed to the surrounding fluid phase, and simultaneously such a supported catalyst became easier to handle and to recover. This catalyst was tested in the CO oxidation reaction and in a sole example of C-C coupling process, the Heck synthesis of butyl cinnamate.

As a part of our ongoing program aimed at finding new eco-efficient synthetic solutions, we decided to broad the scope of Pd-NPs/ZrO_2_ to other important catalytic processes, attempting simultaneously to improve the sustainability of the method through low catalyst loadings, renewable reagents and water as the most desirable green solvent.

This paper reports the application of these favourable conditions to Heck, Ullmann and Suzuki couplings, that are three of the most important Pd-catalysed C-C bond forming reactions employed in chemical, pharmaceutical, and biochemical industries [15].

## 2. Results and Discussion

Preliminary investigations were devoted to find the optimal reaction conditions for all the three coupling processes. To avoid the use of organic cosolvents, a phase transfer agent (PTC) was exploited to facilitate the solvation of low polar starting materials in neat water. In line with our previous findings [16,17,18], the PTC was properly chosen among the quaternary ammonium salts by virtue of their ability to stabilize colloids. Due to the necessity of a base for all the C-C coupling reactions, we decided to use tetra(*n*-butyl)ammonium hydroxide (TBAOH).

Reactions were carried out on a 0.5 mmol scale of the reagents (aryl halides and olefins) dispersed in an aqueous emulsioned mixture (1 mL) of TBAOH (1.5 mmol) and Pd-nanocomposite. The reaction mixture was kept in a small screw cap vial and vigorously stirred under air at the appropriate reaction temperature. 

**Figure 1:**
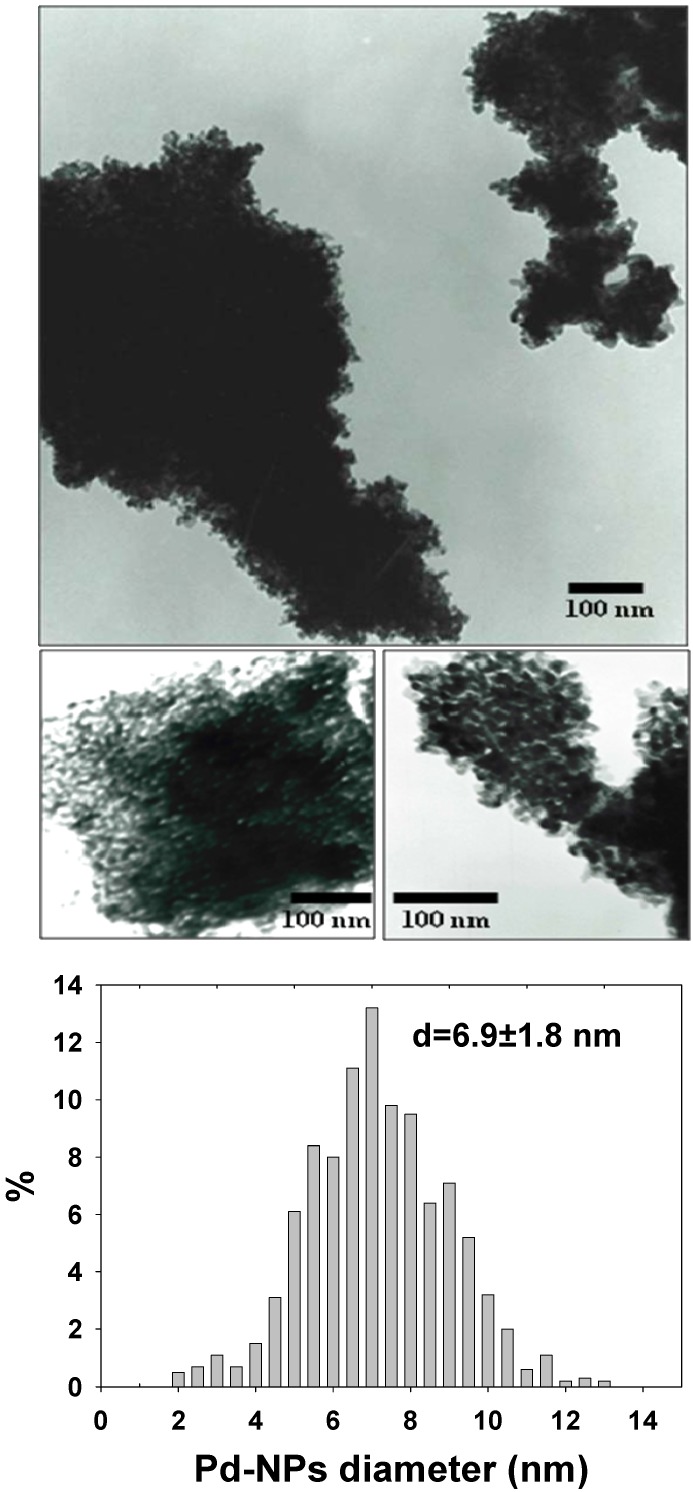
TEM photographs of the Pd-NPs/ZrO_2 _nanocomposite catalyst. The Pd-NPs core diameter size distribution is outlined in the lower panel.

The composite catalyst was truly nanosized, as TEM and morphological analyses revealed that the ZrO_2_ support particle size was 150 ± 70 nm (data not shown, size distribution of as-synthesized ZrO_2_ particles is reported in reference [14] and references therein cited), with the whole surface being covered by spherical Pd nanoparticles, 6.9 ± 1.8 nm in size, and evenly dispersed on the oxide support. Typical TEM micrographs of the nanocomposite catalyst are shown in Figure 1. Pd-NPs can be clearly seen at the borders of the nanocomposite grain, although the whole catalyst surface is fully covered by the small sized Pd nanophases.

To evaluate the catalyst life and the stability of the supported Pd nanoparticles towards release and leaching issues, appropriate recycling experiments were carried out by determining the metal leaching after each run.

### 2.1. Pd/ZrO_2_ nanoparticle- catalysed Heck reaction in water

Palladium-catalysed Heck arylation of olefins is one of the most important carbon-carbon bond-forming processes in synthetic organic chemistry [19,20]. A wide tolerance of functional groups in both reactants allows convenient applications in total synthesis without protecting groups. Due to the industrial importance of this process, the development of cheaper and environmentally benign heterogeneous catalytic systems is very desirable. Pd nanoparticles proved to be highly active in the Heck coupling and a number of supports have been used to prepare stable and recyclable heterogeneous catalysts [11,13,21,22,23,24,25,26] but very few of these couplings have been attempted in water, which is the most desirable eco-friendly solvent.[27]

The coupling of iodobenzene with styrene was initially studied as a model reaction. In a preliminary screen, a reaction temperature of 90 °C, a Pd loading of 0.3 mol %, 2 equiv. of TBAOH and a reaction time of 4 hours were found to be the optimal reaction conditions. Moreover, the replacement of TBAOH with inorganic hydroxides (e.g., KOH) or carbonates (NaHCO_3_ or K_2_CO_3_) gave unsatisfactory results, thus confirming the superiority of tetrabutylammonium hydroxide in this process, due to its ability to simultaneously function as both base and phase transfer agent.

These results also demonstrate that TBAOH plays a special role in creating a favourable environment for the catalyst. Indeed, in the emulsioned mixture generated by the surfactant, reactions presumably occur in the special layer surrounding the nanoparticles’ surface, where a high concentration of the base OH^-^ is reached, with a corresponding increase of the reaction rate. Moreover, besides the role as base, OH^-^ is also expected to behave as ligand, increasing the electron density on palladium that should thus be more active [28].

The recyclability of the supported catalyst in the same model reaction was carefully examined for at least 10 runs. After each run, the supported catalyst was recovered by ultracentrifugation, washed with cyclohexane to remove any adsorbed organic substrates and with water to eliminate the inorganic residues; then the solid was used for the next round without further manipulation (Table 1).

As the results in Table 1 show, the Pd-NPs/ZrO_2_ nanocomposite underwent a slight loss of activity after the third cycle, as indicated by a small decrease in yields and longer reaction times (runs 1–3), but remained stable after that. The yield averaged over ten runs was 72%, with an overall TON of 3,330. In all the cases, small amounts (overall yields <5%) of α-phenylstyrene and the *(Z)*-stilbene were detected by GC-MS.

Following state-of-the-art protocols for trace-element detection (see the Experimental section), the amount of Pd leached from the supported nanocatalyst was determined in all cases by ICP-MS analyses of the supernatant solutions obtained after ultracentrifugation of the reaction mixtures. It is noteworthy that under these conditions, the highest Pd content was found in the supernatant solution of cycle #1, where it was as low as 67 ± 3 ppb (quantified as Pd weight ratio in the supernatant solution).

The mechanism of catalysis by Pd nanoparticles has frequently been disputed in the recent literature. It was proposed that dissolution of palladium species from the surface of the cluster led to the formation of the active species in solution, and the palladium was redeposited onto the colloid after the reaction was finished. The slight loss of catalyst activity after cycles in this case may be due to the loss of palladium from the support during the reaction. Alternatively, it could be due to a simple physical loss of material during the work-up. 

**Table 1:** Recycling experiments on the Heck reaction of iodobenzene and styrene catalysed by Pd-NPs/ZrO_2_ in water^[a]^. 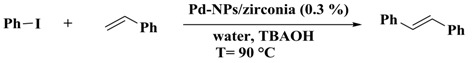

Cycle^[b]^	time (h)^[c]^	Yield(%)^[d]^	Selectivity(%)^[e]^
1	4	81	95
2	4	75	94
3	6	79	95
4	7	68	95
5	7	67	96
6	7	68	94
7	7	70	95
8	7	68	95
9	7	72	97
10	7	70	93

[a] Reaction conditions: iodobenzene (0.5 mmol), styrene (0.65 mmol), TBAOH (1 mmol) and Pd-NPs/ZrO_2_ (Pd 0.4%_w/w_ in the nanocomposite, 0.3 mol % in the reaction mixture) in 1 mL of water were stirred under air for 14 h at 90 °C; [b] for the recycling procedures see the Experimental section; [c] time after which conversion of the starting reagent remained constant; [d] evaluated by GLC using 4,4’-dimethylbiphenyl as external standard (see the Experimental section); [e] percentages, based on the GLC areas, of the *(E)*-stilbene with respect to both the *(Z)* isomer and α-phenylstyrene

**Figure 2:**
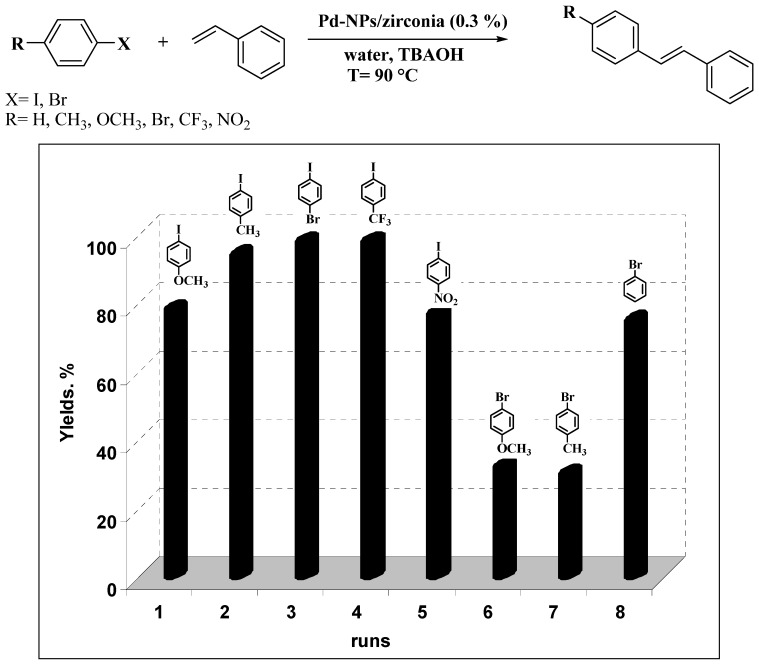
Recycling experiments in the Heck reaction of styrene with a variety of aryl halides catalysed by Pd-NPs/ZrO_2_ in water. Reaction conditions: haloarene (0.5 mmol), styrene (0.65 mmol), TBAOH (1 mmol) and Pd-NPs/ZrO_2_ (Pd 0.3 mol %) in 1 mL of water were stirred under air at 90 °C for 7 hours. The recycling procedure is described in the Experimental section. Yields are evaluated by GLC using biphenyl as external standard. Reactions in runs 6-8 are carried out at 110 °C for 14 hours.

To confirm which one of these two assumptions might be valid, the solid was separated by centrifugation at a conversion value of about 50% in a typical model reaction of iodobenzene and styrene. The supernatant was then allowed to react, affording to an almost negligible increase of conversion of iodobenzene. Considering that: (i) the Pd content in the supernatant is in the low to tens of ppb range and that (ii) in the solid it was 4,000 ppm, based on ICP-MS elemental analysis, these results suggested that negligible Pd leaching takes place during the reaction, and that the catalysis is purely heterogeneous in nature. With these results in hand, a new series of recycling experiments on the Heck arylation of styrene with an array of aryl halides was carried out using a fresh batch of the nanocatalyst (Figure 2).

Both electron-rich and electron-deficient aryl iodides were coupled at 90 °C in moderate to good yield (runs 1–5). Notably, under these conditions 1-bromo-4-iodobenzene was coupled chemoselectively at iodine position (run 3). To activate the less reactive aryl bromides, a higher temperature of 110 °C was used, but under these conditions only the neutral bromobenzene was coupled in a satisfying manner, while the more reluctant electron-rich substrates provided low yields (runs 6–8).

### 2.2. Pd/ZrO_2_ nanoparticle- catalysed Ullmann-type reactions in water using glucose as reductant

In the past decade, biaryls have received increased attention as very important products in the agrochemical and pharmaceutical industries [29]. The core of many types of natural products [30], polymers [31], and ligands for asymmetric catalysis [32], contains the biaryl moiety. Consequently, the development of new and efficient methods of synthesizing these structures is crucial to the work of a great number of organic chemists.

Besides the classic general Ullmann [33,34], Suzuki [35] and Stille [36] coupling reactions, the Pd-catalyzed reductive coupling of haloarenes has gained great attention as a useful route to the formation of aryl-aryl bonds. This simple method for preparing symmetrical biaryls is of ongoing interest as it prevents the use of stoichiometric amounts of expensive or moisture-sensitive organometallic compounds (*i.e.*, boronic acids, stannanes, Grignard reagents *etc*.).

This approach may be particularly attractive for industrial applications providing that it can be carried out in eco-friendly solvents, with easy recyclable catalysts and employing clean and renewable starting materials. From this latter standpoint, in this process the replacement of non-clean or unsafe reducing agents (the most used are zinc powder, formate salts or molecular hydrogen) is mandatory. 

In line with our previous findings [37] on using aldehydes as the reducing agent for the Ullmann-type homocoupling of bromoarenes, we decide to exploit sugars (renewable reagents) as reductants in the process catalysed by our highly recyclable supported Pd nanocatalysts which is suitable to be used in aqueous medium.

Although several reductive aryl-aryl coupling protocols have been studied in water [13], the use of either free or supported Pd nanoparticles as the catalyst in this process is a relatively unexplored area [10,19]. In addition, no examples have been reported on the use of sugars as stoichiometric reductants. Among several monosaccharides, we focused our attention particularly on glucose, which is known to smoothly reduce some metals in colloidal form [38,39].

In line with this hypothesis, we begun to test the activity of Pd-NPs/ZrO_2_ by comparing the performances of glucose with those of the most common reductants used in this process (Table 2). To this end, a series of model reductive couplings of bromobenzene were carried out under conditions very close to those of the previous Heck reaction, that is PhBr 0.5 mmol, TBAOH 1.5 mmol, Pd source 0.5 mol %, at 90 °C in water as the solvent.

**Table 2:** Screening of reductants in the Ullmann-type homocoupling of bromobenzene catalysed by Pd-NPs/ZrO_2_^[a]^. 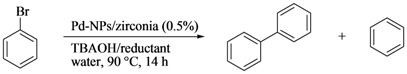

Run	Reductant	Yields^[b]^ (%)	Selectivity^[c]^ (%)
1	Glucose	80	96
2	Fructose	50	68
3	Hydroquinone	10	25
4	Zn powder	33	39
5	NaBH_4_	40	5
6	HCOONa	85	30
7	Propanal	20	50
8	Glyceraldehyde	70	65
9	Ascorbic acid	85	95
10	Glucose^[d]^	85	98
11	Glucose^[e]^	86	50

[a] Reaction conditions: bromobenzene (0.5 mmol), reductant (0.5 mmol), TBAOH (1.5 mmol) and Pd nanocomposite (0.5 mol %) in 1 mL of H_2_O, heated under stirring at 90 °C for 14 hours; [b] yields based on GLC areas by using 4,4’-dimethylbiphenyl as an external standard; [c] percentage of the coupling respect than the reduction product (benzene); [d] with 0.25 mmol of glucose; [e] With 1 mmol of glucose.

The results in Table 2 show that, among the reported reductants, ascorbic acid was the sole reagent capable of providing yields and selectivity comparable with those of glucose (Table 2, runs 1–9). Interestingly, due to the capacity of glucose to provide many reducing equivalents, the coupling could be accomplished with both good yields and selectivity also in the presence of a half equimolar amounts of reductant (0.25 mmol, Table 2, run 10). On the contrary, with an excess amounts of glucose (1 mmol), selectivity towards the homocoupling product was drastically lowered (Table 2, run 11).

**Figure 3:**
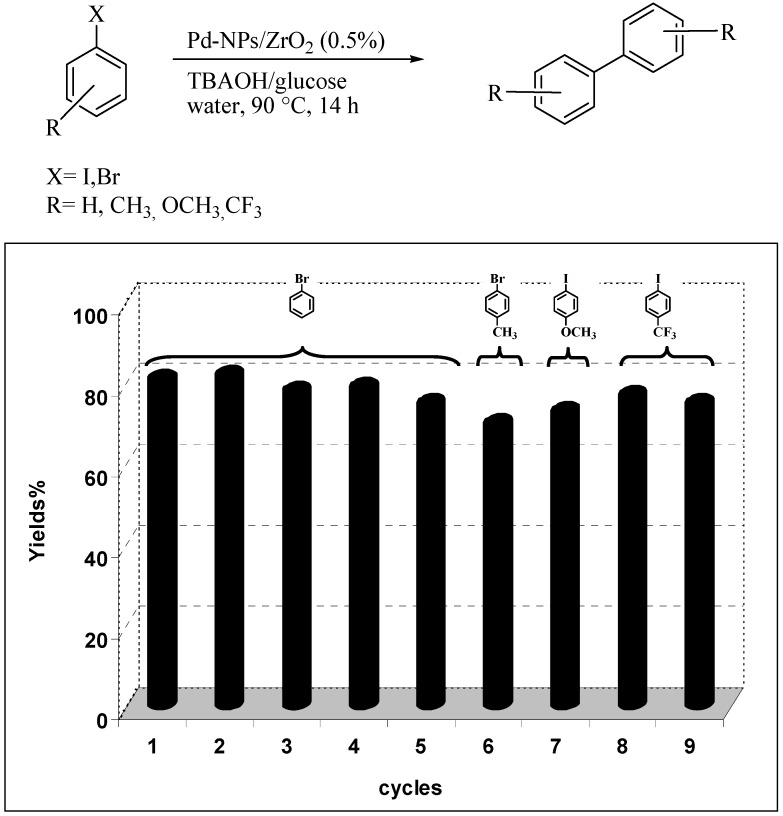
Recycling experiments in the Ullmann-type homocoupling of aryl halides catalysed by Pd-NPs/ZrO_2_ in water. Reaction conditions: haloarene (0.5 mmol), TBAOH (1 mmol) and Pd-NPs/ZrO_2_ (Pd 0.5 mol %) in 1 mL of water were stirred under air at 90 °C for 14 hours. For the recycling procedure see the Experimental section. Yields are evaluated by GLC using the proper substituted biphenyl as external standard.

In Figure 3 we have plotted the recycling efficiencies in the reductive homocoupling of bromobenzene and three other substituted halobenzenes bearing either an electron-rich or an electron-poor substituent. The resulting column graph shows that the catalyst retained its high catalytic activity of over nine consecutive repeated cycles.

### 2.3. Pd/ZrO_2_ nanoparticle-catalysed Suzuki coupling

The Suzuki–Miyaura reaction is an efficient method for preparing unsymmetrical biaryls from an aryl halide and boronic acid with tolerance towards a wide range of substituents. Palladium nanoparticles have been considered as the catalyst in a number of reports [40,41,42], and for their immobilization solid supports such as polyaniline nanofibers [10], MgO [43], alumina-based oxides [44], diatomite [25] and double-layered hydroxide (LDH) [28] have also been used.

The Suzuki cross-coupling reactions with our supported catalyst were carried out in water at 90 °C using aryl bromides and iodides as substrates and phenyl boronic acids as nucleophiles. Also in this case, reaction conditions were chosen after a series of initial experiments carried out at different temperatures and with different bases. Among the latter, TBAOH was confirmed as the most efficient. In addition, the aqueous medium proved to have a positive effect on the reaction rate probably because of its ability to dissolve boron side products coating the catalyst surface. 

**Table 3:** Recycling experiments on the Suzuki reaction catalyzed by Pd-NPs/ZrO_2_ in water^[a]^. 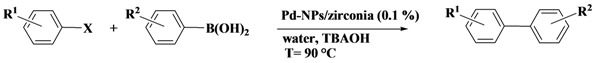

Cycle^[b]^	X	R^1^	R^2^	Product	Yield (%)^[c]^
1	I	H	H	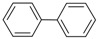	90
2	I	H	OCH_3_	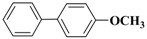	88
3	I	OCH_3_	H	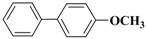	75
4	I	CH_3_	H	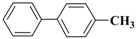	84
5	I	NO_2_	H	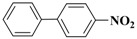	78
6	Br	H	H	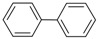	75
7	Br	H	OCH_3_	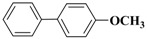	88
8	Br	C(O)CH_3_	H	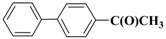	90
9	Br	OCH_3_	H	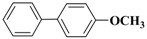	40
10	Br	H	CH_3_	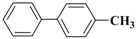	79

[a] Reaction conditions: aryl halide (0.5 mmol), boronic acid (0.65 mmol), TBAOH (1 mmol) and Pd-NPs/ZrO_2_ (Pd 0.1 mol %) in 1 mL of water were stirred under air at 90 °C for 14 hours; [b] for the recycling experiments see the Experimental section; [c] evaluated by GLC using 4,4’-dimethyl-biphenyl as external standard (see Experimental section)

Results presented in Table 3 show that also in these cross-coupling reactions the palladium supported material could be recycled up to ten times without any appreciable loss of activity. Indeed, an average yield of 83% was found for a number of aryl bromides, with the exception of electron-rich 4-bromoanisole, which proved to be less reactive (Table 3, cycle 9). As already found in the previous experiments, no significant quantities of Pd (≤67 ± 3 ppb) were detected in solution after the last reaction, confirming the stability of our zirconia supported Pd material.

## 3. Experimental

### 3.1. General

The synthesis of the Pd/ZrO_2_ nanocomposite has been described in Ref. [14]. X-ray Diffraction (XRD) and X-ray Photoelectron Spectoscopy (XPS) were used respectively to assess the structure and chemical composition of the nanomaterials. XRD confirmed the tetragonal crystalline structure of the ZrO_2_ nanostructured support. XPS was used to check the ceramic oxide surface stoichiometry (that was always close to 2.0), as well as the Pd surface availability and chemical speciation. Pd surface atomic percentage was found to be comprised in the range between 0.2 and 0.4%_at_, as a function of the conditions employed during the nanocatalyst electrochemical synthesis. Moreover, XPS revealed the presence of two chemical environments on the catalyst surface, namely Pd(II) and nano-sized Pd(0) [14], their abundance ratio being close to 1/3 in the as-prepared composite.

The bulk Pd concentration in the nanocomposite was quantified by Inductively Coupled Plasma Mass Spectrometry (ICP-MS). Preliminarily to ICP-MS analyses, samples were subjected to a complete mineralization/dissolution process, as follows. Aliquots of about 10 mg of ZrO_2_ and Pd/ZrO_2_ were dissolved by acid attack with a solution of 95–97% H_2_SO_4_ (Merck trace selected for trace analysis reagent) and 67% HNO_3_ (Normatom trace selected for trace analysis reagent) in 1:1 (v/v) ratio, using open Teflon vessels until dryness. The residues were re-dissolved in 67% HNO_3_ and made up to a final 2% volume with deionized water.

The ultracentrifuged-supernatant solution coming from the first cycle of Heck reaction, after dryness, was subjected to the same process. Elemental analysis were carried out on dissolved samples, supernatant solution and mineralization-blank using an ICP-MS Elan 9000 Spectrometer (Perkin Elmer). Indium was used as internal standard and ^105^Pd element was determined in each sample. The choice of 105-mass represents the optimum arrangement between natural abundance (^105^Pd = 22.33%) and possible interferences (Sr and Y oxides). The Pd content into the supported catalyst was 0.38 ± 0.01%_w/w_; the highest amount of leached Pd (occurring at the first cycle of Heck reaction) was 67 ± 3 ppb into the supernatant solution, corresponding to 0,06% of the starting palladium amount added as catalyst to the pristine reaction mixture. Such an evidence is noteworthy, since it clearly supports the highly recyclable character of the nanocatalyst.

Reactions were monitored by GCL and GC-MS techniques by using an Agilent 5890A gas chromatograph (equipped with a FID) and an Agilent 6850/MD 5975C instrument, respectively. Both instruments were equipped with a capillary column HP-5MS (Agilent, l. 30 m, i.d. 0.25 mm, s.p.t. 0.25 μ) and analyses were carried out with the following temperatures program: T_i_ = 80 °C, t_1_ = 3 min, rate = 25 °C/min, T_f_ = 280 °C, flow rate = 1 mL/min.

### 3.2. Representative procedure for Pd-NPs/ZrO_2_-catalyzed Heck coupling

Iodobenzene (0.5 mmol), styrene (0.5 mmol), TBAOH (1.5 mmol), Pd-NPs/ZrO_2_ (0.3 mol %) and H_2_O (1 mL) were placed in a 1 mL gas-tight vial, equipped with a screw cap and a magnetic bar. The flask was heated under air and stirring at the appropriate reaction temperature for a maximum of 14 hours (see Table 1 and Figure 2). Then, cyclohexane (3 mL) was added to the reaction mixture and, after centrifugation, the organic layer was separated from the aqueous phase. These operations were repeated three times and then a known amount of an external standard (4,4’-biphenyl) was added to the collected organic layers and they were examined by GLC and GC-MS, providing both yields and product identification by comparison with spectral data reported in the literature. The residue containing the catalyst was washed twice with water (3 mL) and each time, after centrifugation, the aqueous phase was removed. Finally, fresh reagents were added to the remaining solid and the mixture was allowed to react for another run (see Table 3).

### 3.3. Representative procedure for Pd-NPs/ZrO_2_-catalyzed Ullmann-type homocoupling

Bromobenzene (0.5 mmol), glucose (0.25 mmol), TBAOH (1.5 mmol), Pd-NPs/ZrO_2_ (0.5 mol %) and H_2_O (1 mL) were placed in a 1 mL gas-tight vial, equipped with a screw cap and a magnetic stir bar. The flask was heated under air and under stirring at the reaction temperature for a maximum of 14 hours (see Table 2 and Figure 3). Then, the reaction mixture was treated with cyclohexane (3 mL) and, after centrifugation, the organic layer was separated from the aqueous phase. These operations were repeated three times and a known amount of an external standard (4,4’-dimethylbiphenyl) was then added to the collected organic layers which were examined by GLC and GC-MS, providing both yields and product identification by comparison with spectral data reported in the literature. The residue containing the catalyst was washed twice with water (3 mL) and each time, after centrifugation, the aqueous phase was removed. Finally, fresh reagents were added to the remaining solid and the mixture was allowed to react for another run.

### 3.4. Representative procedure for Pd-NPs/ZrO_2_-catalyzed Suzuki coupling

Haloarene (0.5 mmol), phenylboronic acid (0.75 mmol), TBAOH (1.5 mmol), Pd-NPs/ZrO_2_ (0.5 mol %) and H_2_O (1 mL) were placed in a 1 mL gas-tight vial, equipped with a screw cap and a magnetic stir bar. The flask was heated under air and under stirring at the appropriate reaction temperature for a maximum of 14 hours (see Table 3). Then, cyclohexane (3 mL) was added to the reaction mixture and after centrifugation the organic layer was separated from the aqueous phase. These operations were repeated three times, and then a known amount of an external standard (a suitable substituted biphenyl) was added to the collected organic layers and they were examined by GLC and GC-MS, providing both yields and product identification by comparison with spectral data reported in the literature. The residue containing the catalyst was washed twice with water (3 mL) and each time, after centrifugation, the aqueous phase was removed. Finally, fresh reagents were added to the remaining solid and the mixture was allowed to react for another run.

## 4. Conclusions

In summary, we have demonstrated that our composite resulting from the electrochemical impregnation of Pd nanoparticles on a tetragonal ZrO_2_ nanopowder, can be used as a suitable heterogeneous catalyst in three important Pd catalysed C-C coupling processes such as Heck, Ullmann and Suzuki reactions. Very sustainable conditions were used such as aqueous media and clean and renewable reagents. The use of tetrabutylammonium hydroxide (TBAOH) as base and phase transfer catalyst, generating an emulsioned reaction mixture, allowed to overcome the solubility problems of the low polar starting materials in neat water.

High activity and good TONs (>3 × 10^3^) were achieved for the Heck reaction of iodobenzene and styrene within 7 h, and the catalyst can be recovered easily and reused many times. Analogous to homogeneous Heck catalysis, the gradation of the aryl bromide conversion depended on the electronic effect of substituent in *para*-position to the bromine. The studies also revealed that the palladium leaching into the solution during the reaction is negligible and therefore the catalysis is heterogeneous in nature.

Remarkably advantageous conditions were found to perform the Ullmann-type reductive coupling of an array of iodo- and bromoarenes. In particular, the use of glucose (a renewable biomass product), in sub-stoichiometric amounts, provides the method with great benefits in terms of safety, economy and sustainability, especially if compared with the most known protocols which make use of non-clean or unsafe reducing agents such as Zn powder, formate salts or molecular hydrogen.

Several Suzuki couplings of aryl iodides and bromides were carried out under very similar conditions. In this case the aqueous medium proved to be particularly advantageous for its ability to dissolve boron side products coating the catalyst surface. Also in this case, the palladium supported material was found to be reusable up to ten times without an appreciable loss of activity.

Therefore, considering that our heterogeneous catalyst proved to be efficient at low loadings and highly recyclable (al least ten times) in a number of important C-C coupling processes carried out in eco-friendly conditions, we believe that it can compete with the most efficient known protocols, and due to its simple operating procedure we can anticipate that it will find wide applicability.
